# One‐step and sequential SARSCOV‐2 polymerase chain reaction tests would not work every time

**DOI:** 10.1002/jcla.24226

**Published:** 2022-01-08

**Authors:** Firouze Hatami, Mohammad Mahdi Rabiei, Farid Javandoust Gharehbagh, Mohamad Amin Pourhoseingholi, Shahram Sabeti, Mahnaz Kheyrian, Ilad Alavi Darazam

**Affiliations:** ^1^ Department of Infectious Diseases and Tropical Medicine Loghman Hakim Hospital Shahid Beheshti University of Medical Sciences Tehran Iran; ^2^ Infectious Diseases and Tropical Medicine Research Center Shahid Beheshti University of Medical Sciences Tehran Iran; ^3^ Gastroenterology and Liver Diseases Research Center Research Institute for Gastroenterology and Liver Diseases Shahid Beheshti University of Medical Sciences Tehran Iran; ^4^ Pathology Ward Loghman Hakim Hospital Shahid Beheshti University of Medical Sciences Tehran Iran

**Keywords:** COVID‐19, repeat positivity/negativity, RT‐PCR

## Abstract

**Introduction:**

RT‐PCR is widely used as a diagnostic test for the detection of SARS‐CoV‐2. In this study, we aim to describe the clinical utility of serial PCR testing in the final detection of COVID‐19.

**Method:**

We collected multiple nasopharyngeal swab samples from patients who had negative RT‐PCR test on the first day after hospitalization. RT‐PCR tests were performed on the second day for all patients with initial negative result. For the patients with secondary negative results on day 2, tertiary RT‐PCR tests were performed on day 3 after hospitalization.

**Result:**

Among 68 patients with initial negative test results, at the end of follow‐up, the mortality number was 20 (29.4%). About 33.8% of patients had subsequent positive PCR test results for the second time and 17.4% of the patients who performed third PCR test had positive result.

**Conclusion:**

Based on this study, serial RT‐PCR testing is unlikely to yield additional information.

## Graphical abstract

Among 68 patients with initial negative test results, 33.8% of patients had subsequent positive PCR test results for second time and 17.4% of the patients who performed third PCR test had positive result. Performing serial RT‐PCR tests could not significantly increase the detection capability of SARS‐COV‐2 after initial negative test results. Repeated RT‐PCR tests in short time intervals for patients with initial negative results also remained negative.

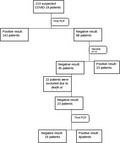

## 1 INTRODUCTION

Emergence of Coronavirus Disease 2019 (COVID‐19) caused by severe acute respiratory syndrome coronavirus‐2 (SARS‐CoV‐2) exerts myriads of harmful consequences on healthcare systems.^1^ Regarding the high contagion rate of COVID‐19 as well as the absence of specific therapeutic drugs, timely detection of the disease is important in order to control the sources of infection and prevent the illness progression.

Due to non‐specific signs and symptoms of COVID‐19, implementing of diagnostic tests based on detection of the viral sequence by real‐time reverse transcription‐polymerase chain reaction assay (RT‐PCR) has been a robust technique to confirm the infection.^2^ RT‐PCR is a specific and simple qualitative assay; hence, diagnostic test for COVID‐19^3^ is of great interest. Nevertheless, eliciting of false negative and false positive results is probable using this technique.^4^

The result of SARS‐CoV‐2 PCR is highly dependent on sampling time and specimen type.^5^ In a study, many suspected cases, in spite of exhibiting clinical characteristics and typical radiologic findings for COVID‐19, were not diagnosed by RT‐PCR.^6^ Regular Assessment of sensitivity and specificity of tests by Food and Drug Administration (FDA) and clinical researchers in pandemic era is required to avoid confusion in ruling in/out the infection.

Some clinicians suggested that performing serial RT‐PCR tests on suspected cases with initial negative RT‐PCR test improves detection capability. To date, there is no recommendation on the efficacy of serial testing in patients with an initial negative PCR. In this study, our aim was to explore the clinical utility of serial PCR testing and the contribution of each RT‐PCR test to the final detection of COVID‐19.

## 2 METHOD

### 2.1 Patient population

In this study, confirmed COVID‐19 patients with clinical manifestations, positive CT‐scan results, and/or consecutive negative RT‐PCR tests in a short period of time were included. All the participants were in intensive care unit and the symptoms of COVID‐19 were classified as moderate to severe based on the guideline.^7^ The study was confirmed by the Ethics in Medical Research Committee IR.SBMU.RETECH.REC.1399.033.

### 2.2 Sample collection

Nasopharyngeal swab samples were collected on the second day after hospitalization from patients who had negative RT‐PCR test on the first day after hospitalization. RT‐PCR tests were performed on the second day for all patients with initial negative result. For the patients with secondary negative results on day 2, tertiary RT‐PCR tests were performed on day 3 after hospitalization at Loghman Hakim hospital as a major referral center. The personnel collecting the samples and performing tests were the same in each of the tests.

Synthetic fiber swabs on a wire shaft and sterile tubes containing Viral Transport Medium (VTM) were used to collect nasopharyngeal specimens. Prior to specimen collection, tubes were labeled with patients' information. Nasopharyngeal Swabs were collected according to Centers for disease control and prevention (CDC) recommendations (https://www.cdc.gov/coronavirus/2019‐ncov/lab/guidelines‐clinical‐specimens.html). Swabs were placed into VTM and were kept at 2–8°C for less than 24 h.

### 2.3 RNA extraction and RT‐PCR

Extraction procedure was carried out using commercial extraction kit (ROJE) according to manufacturer's instructions. Liferiver real‐time multiplex RT‐PCR kit was used for qualitative detection of COVID‐19. The kit contains super mix, enzyme mix, negative control (NC), positive control (PC), and internal control (IC). The IC was added into extraction mixture 1 µl/test. The NC was extracted with the same protocol as for the samples. Master mix (20 µl consisting of 19 µl of super mix and 1 µl of enzyme mix) was added to each RT‐PCR reaction tube. Then, 5 µl of nucleic acid extracted from NC and specimen and PC (without extraction) were added to separate reaction tubes. PCR was performed at 45°C for 10 min, 95°C for 3 min, 95°C for 15 s, and 58°C for 30 s for 45 cycles. Fluorimeter channels including FAM (for ORF1ab), HEX (for gene N), and Cal Red 610 (for Gene E) were used for the detection of amplified virus DNA fragment. The IC was detected at Cy5 channel.

### 2.4 Results interpretation

#### 2.4.1 Positive results for SARS‐COV‐2

Positive signals (Ct ≤ 41) were detected in FAM, HEX, and Cal Red 610 channels or in FAM and HEX or in FAM and Cal red 610 channels.

#### 2.4.2 Negative results for SARS‐COV‐2

Negative signals (Ct > 41) were found in FAM, HEX, and Cal Red 610 channels and positive signal (Ct < 41) was observed in Cy5 channel.

#### 2.4.3 Inconclusive results

Only one channel from FAM, HEX, and Cal Red 610 was detected with Ct ≤ 41; positive signals were detected in HEX and Cal red 610 and negative signal (Ct > 41) was found in FAM channel. Inconclusive results were repeated to obtain positive or negative results. Therefore, our final results just included positive or negative results.

#### 2.4.4 Invalid results

Values of Ct > 41 or no value was measured in the fluorescence channels FAM, HEX, and Cal Red and values of Ct ≥ 41 or no value was measured in the channel Cy5. Invalid results were also repeated.

### 2.5 Statistical analysis

Continuous variables were described using mean, median, and interquartile range (IQR) values and categorical variables were described as frequency rates and percentages, and Chi‐square test or Fisher exact test was used to compare the distribution of categorical data including demographic and clinical variables between patients with negative PCR test and positive PCR test. *p*‐Value less than 0.05 was considered as statistically significant. All statistical analyses were performed using R version 3.6.1 software.

## 3 RESULTS

Out of 210 suspected COVID‐19 patients with clinical manifestations and positive CT results, 68 patients (32%) had initial negative RT‐PCR tests. Among 68 participants, the distribution of males (age 64.1 ± 15.4) was 36 (52.9%). The mean time between the onset of clinical manifestation and collected first sample was 9.4 ± 5.0 days. At the end of follow‐up, mortality number was 20 (29.4%). Demography, clinical symptoms, and background of diseases were outlined in Table 1. The most frequent symptom observed among patients at the admission time was myalgia followed by cough and hypoxia, respectively. Hypertension, diabetes, and pulmonary disease were the most common comorbidities in these patients (Table 1).

**TABLE 1:** Baseline demographic and clinical characteristics of patients infected with COVID‐19 with negative PCR test for first round (comparing results of second PCR test)

Variables	Total (n = 68)	Negative PCR test (n = 45)	Positive PCR test (n = 23)
Age			
Mean ± SD	64.1 ± 15.4	65.2 ± 16.3	62.1 ± 13.4
Gender No (%)			
Male	36 (52.9)	22 (48.9)	14 (60.9)
Symptoms No (%)			
Fever	30 (45.5)	19 (44.2)	11 (47.8)
Cough	50 (75.8)	29 (67.4)	21 (91.3)
Hypoxia	48 (73.8)	31 (72.1)	17 (77.3)
Myalgia	52 (78.8)	32 (74.4)	20 (87.0)
Headache	13 (19.7)	10 (23.3)	3 (13.0)
Chills	20 (30.8)	14 (32.6)	6 (27.3)
Shortness of breath	49 (74.2)	31 (72.1)	18 (78.3)
Sore throat	20 (30.3)	14 (32.6)	6 (26.1)
Diarrhea	11 (16.7)	7 (16.3)	4 (17.4)
First oxygen saturation			
Median (IQR)	86 (88–88.5)	85 (78–89)	87 (85–88.2)
Mortality No (%)	20 (29.4)	12 (26.7)	8 (34.8)
The type of comorbidities No (%)			
Diabetes	20 (29.4)	9 (20.0)	11 (47.8)
Hypertension	26 (38.2)	17 (37.8)	9 (39.1)
Pulmonary disease	12 (17.6)	8 (17.8)	4 (17.4)

Secondary RT‐PCR test for the patients (n = 68) was still negative in 45 patients (66.2% of all negative ones and 21% of the all patients) and positive in 23 patients (33.8% of all negative ones). The third RT‐PCR test was conducted for patients with secondary negative RT‐PCR result with the exception of those who were expired or discharged (n = 23). From the total 23 participants, 19 were still negative (82.6% of participants with secondary negative RT‐PCR test) and just 4 patients had positive PCR test (17.4%).

## 4 DISCUSSION

In this study, performing serial RT‐PCR tests could not significantly increase the detection capability of SARS‐COV‐2 after initial negative test results. Based on our findings in most cases, repeated RT‐PCR tests in short time intervals for patients with initial negative results also remained negative. The lack of promising laboratory tests for timely detection of SARS‐COV‐2 is tangible. Eliciting false positive results can be seen even by employing various SARS‐COV‐2 molecular detection kits.

RT‐PCR sensitivity is highly affected by improper performing of laboratory practice standards and personal skills.^4^ Proper specimen collection is an essential step for virus detection; it minimizes false negative results. Therefore, specimen collection, sample preparation, and experiments should be performed by well‐trained staffs.^8^ Considering these limitations as an important issue, frequent false negative RT‐PCR results are reviewed. In our study, in order to adjust the impact of specimen collection and laboratory practice, the personnel collecting the samples and performing tests were the same in each of the tests since our aim was to evaluate the contribution of each RT‐PCR test to the final detection of COVID‐19.

High false negative results can be expected because of the emergence of various variants of SARS‐COV‐2 in each population.^9^ Mutations occur frequently in SARS‐COV‐2, and the RT‐PCR method usually detects 2 or 3 genes of SARS‐CoV2.^10^ Therefore, false negative is a disadvantage of RT‐PCR, and even performing serial RT‐PCR tests could not improve its detection capability.

Due to some clinical factors, repeated RT‐PCR tests are preferred in some cases with suspected COVID‐19. The viral load appears to peak approximately 24 h before the onset of symptoms in the upper respiratory tract and then decreases over the next 5 days.^11^ The severity of the COVID‐19 infection also plays a major role, and severe cases have a higher viral load.^12^ Also, inappropriate specimen collection, handling, and processing may increase the false negative results.^13^ Therefore, some healthcare providers repeat the RT‐PCR test after initial negative result test in patients with high clinical suspicion of COVID‐19. A study reported that only 21.4% of COVID‐19 patients had positive test results on their third consecutive test after two negative results.^14^ In a multicenter cohort study, repeated RT‐PCR tests were performed within 7 days among patients with initial negative test results. Only 2.0% of patients had subsequent positive PCR test.^15^ This study indicates that repeated RT‐PCR could not provide additional information. Another study demonstrated that almost 25% of SARS‐CoV‐2 positive patients had a negative result in initial testing.^16^ Based on a case report, the initial RT‐PCR result was negative for a patient. Four days later, positive result was reported for the patient. The third RT‐PCR performed 4 days after second test was surprisingly negative.^17^ The fact that early sampling minimizes false negative RT‐PCR result should be considered. The best time to obtain an upper respiratory specimen is the early phase of the disease course when the viral replication is high.^18^ Albeit RT‐PCR is rapid, sensitive, and specific, each molecular result should be interpreted individually for each case according to the clinical manifestation because there is the risk of false negative of RT‐PCR for some reasons.

The sensitivity and specificity of a diagnostic test are evaluated based on a gold standard test. Although several pitfalls, low specificity, and sensitivity have been mentioned for RT‐PCR, almost all global and international and national guidelines rely on this technique as the gold standard test for SARS‐COV‐2 detection. Thus, absence of a reliable gold standard has made it difficult to evaluate RT‐PCR accuracy. Evaluation of other tests for diagnosing COVID‐19 as well as definition of confirmed, probable, and possible cases according to the results of RT‐PCR is not a reliable approach in order to use it for management, screening, and surveillance. Ongoing studies to estimate the sensitivity and specificity of COVID‐19 PCR assays will help clinicians to determine the positive and negative predictive values depending on disease burden in their area.

Although there are no recommendations for serial testing, IDSA has a recommendation for patients with a negative initial PCR test. The IDSA recommended that repeat testing should be done24–48 h after initial testing and once the initial NAAT result has turned negative. Another specimen type, preferably a lower respiratory tract specimen if the patient has signs/symptoms of lower respiratory tract infection, should be considered for repeat testing.^19^

The limitation of our study was that we did not use a lower respiratory tract specimen in patients with signs of lower respiratory tract infection.

## 5 CONCLUSION

Among 68 patients with initial negative test results, 33.8% of patients had subsequent positive PCR test results for second time and 17.4% of the patients who performed third PCR test had positive result. Serial RT‐PCR testing is unlikely to yield significantly additional clinical information; however, the second test could be performed because the false negative rate was very high in the single test. Nevertheless, the decision to repeat testing must be more tactful.

## CONFLICT OF INTEREST

We declare no competing interests.

## Data Availability

The datasets generated during and/or analyzed during the current study are available from the corresponding author on reasonable request.

## References

[1] Matheson NJ , Lehner PJ . How does SARS‐CoV‐2 cause COVID‐19? Science. 2020;369(6503):510‐511. 32732413 10.1126/science.abc6156

[2] Shen M , Zhou Y , Ye J , et al. Recent advances and perspectives of nucleic acid detection for coronavirus. J Pharm Anal. 2020;10(2):97‐101. 32292623 10.1016/j.jpha.2020.02.010 PMC7102540

[3] Wan Z , Yn Z , He Z , et al. A melting curve‐based multiplex RT‐qPCR assay for simultaneous detection of four human coronaviruses. Int J Mol Sci. 2016;17(11):1880. 10.3390/ijms17111880 PMC5133880 27886052

[4] Tahamtan A , Ardebili A . Real‐time RT‐PCR in COVID‐19 detection: issues affecting the results. Expert Rev Mol Diagn. 2020;20(5):453‐454. 32297805 10.1080/14737159.2020.1757437 PMC7189409

[5] Binnicker MJ . Challenges and controversies to testing for COVID‐19. J Clin Microbiol. 2020;58(11):e01695‐20. 32817231 10.1128/JCM.01695-20 PMC7587118

[6] Wang Y , Kang H , Liu X , Tong Z . Combination of RT‐qPCR testing and clinical features for diagnosis of COVID‐19 facilitates management of SARS‐CoV‐2 outbreak. J Med Virol. 2020;92(6):538‐539. 32096564 10.1002/jmv.25721 PMC7233289

[7] National Institutes of Health (NIH) Clinical presentation of people with SARS‐CoV‐2 infection. Available from: https://www.covid19treatmentguidelines.nih.gov/overview/clinical‐presentation/. Accessed October 19, 2021.

[8] Yang Y , Yang M , Yuan J , et al. Laboratory diagnosis and monitoring the viral shedding of SARS‐CoV‐2 Infection. Innovation (N Y). 2020;1(3):100061. 33169119 10.1016/j.xinn.2020.100061 PMC7609236

[9] Genetic variants of SARS‐CoV‐2 may lead to false negative results with molecular tests for detection of SARS‐CoV‐2 – Letter to Clinical Laboratory Staff and Health Care Providers 2021. Available from https://www.fda.gov/medical‐devices/letters‐health‐care‐providers/genetic‐variants‐sars‐cov‐2‐may‐lead‐false‐negative‐results‐molecular‐tests‐detection‐sars‐cov‐2. Accessed October 19, 2021.

[10] Chang MC , Hur J , Park D . Interpreting the COVID‐19 test results: a guide for physiatrists. Am J Phys Med Rehabil. 2020;99(7):583‐585. 32427600 10.1097/PHM.0000000000001471 PMC7268832

[11] He X , Lau EH , Wu P , et al. Temporal dynamics in viral shedding and transmissibility of COVID‐19. Nat Med. 2020;26(5):672‐675. 32296168 10.1038/s41591-020-0869-5

[12] Liu Y , Yan L‐M , Wan L , et al. Viral dynamics in mild and severe cases of COVID‐19. Lancet Infect Dis. 2020;20(6):656‐657. 32199493 10.1016/S1473-3099(20)30232-2 PMC7158902

[13] Woloshin S , Patel N , Kesselheim AS . False negative tests for SARS‐CoV‐2 infection—challenges and implications. N Engl J Med. 2020;383(6):e38. 32502334 10.1056/NEJMp2015897

[14] Xiao AT , Tong YX , Zhang S . False negative of RT‐PCR and prolonged nucleic acid conversion in COVID‐19: Rather than recurrence. J Med Virol. 2020;92(10):1755‐1756. 32270882 10.1002/jmv.25855 PMC7262304

[15] Challener DW , Shah A , O'Horo JC , Berbari E , Binnicker MJ , Tande AJ . Low utility of repeat real‐time pcr testing for SARS‐CoV‐2 in clinical specimens. Mayo Clin Proc. 2020;95(9):1942‐1945. Elsevier. 32861337 10.1016/j.mayocp.2020.06.020 PMC7334934

[16] Li Y , Yao L , Li J , et al. Stability issues of RT‐PCR testing of SARS‐CoV‐2 for hospitalized patients clinically diagnosed with COVID‐19. J Med Virol. 2020;92(7):903‐908. 32219885 10.1002/jmv.25786 PMC7228231

[17] Kanamoto M , Tobe M , Takazawa T , Saito S . COVID‐19 with repeated positive test results for SARS‐CoV‐2 by PCR and then negative test results twice during intensive care: a case report. J Med Case Rep. 2020;14(1):1‐4. 33028403 10.1186/s13256-020-02534-2 PMC7538677

[18] Zou L , Ruan F , Huang M , et al. SARS‐CoV‐2 viral load in upper respiratory specimens of infected patients. N Engl J Med. 2020;382(12):1177‐1179. 32074444 10.1056/NEJMc2001737 PMC7121626

[19] IDSA guidelines on the diagnosis of COVID‐19: serologic testing 2020.

